# Mental health status and stressful life events among postgraduate nursing students in Cyprus: a cross-sectional descriptive correlational study

**DOI:** 10.1186/s12912-023-01463-x

**Published:** 2023-08-30

**Authors:** Sokratis Sokratous, Giorgos Alexandrou, Rafailia Zavrou, Maria Karanikola

**Affiliations:** 1 Department of Nursing, Faculty of Health Sciences, Cyprus University of Technology, Limassol, Cyprus; 2 Mental health Services, Nicosia, Cyprus; 3 Mental health Services, Paphos, Cyprus

**Keywords:** Mental health status, University students, Mental distress symptoms, Stressful life events

## Abstract

**Background:**

Despite prior evidence supporting the association between stressful life events and mental health status, there are limited data on the number and severity of stressful life events and their effects on university students’ mental health status. Therefore, the present study aimed to: (a) examine mental health status and subsequent predictors of clinically significant mental distress symptoms, (b) estimate the number and severity of stressful life events, and (c) explore the associations between mental health status, stressful life events (number and severity) and sociodemographic characteristics.

**Methods:**

This study was descriptive, cross-sectional, and correlational with internal comparisons. A convenience sample of 90 Master of Science in nursing and midwifery students, participated in the study. Participants with a General Health Questionnaire-28 (GHQ-28) total score ≥ 24 were considered to have clinically significant mental distress symptoms. Descriptive and inferential statistics were evaluated, and Pearson’s chi-square test for group differences was used to analyse the data. Analysis of variance and t-tests were used for comparisons between two or more groups, and regression analyses were employed to identify the predictors of GHQ-28 scores and clinical mental distress symptoms.

**Results:**

The final sample comprised 90 students (response rate: 97.8%), 33 (36.7%) of whom reported clinically significant symptoms of mental distress. Students with divorced parents [29.92 (± 10.62), *p < .05*] scored high on the GHQ-28. Participants who had low/no satisfaction with the education system posted higher scores than participants who had high/very high satisfaction [24.82 (± 11.68) vs. 17.93 (± 9.78), *p < .05*]. In the subscale measuring depressive symptoms, there was a statistically significant gender difference, with men reporting higher scores than females. [3.0± (3.69) vs. 1.60 (± 2.42), *p* = .034]. In multiple analyses of sociodemographic characteristics and those who scored higher on the Life Events Scale for Students (LESS) scale (≥ 340), the loss of parent/s was associated with the total GHQ-28 score (B=-17.046, *p < .001).* In multiple analyses, chronic physical disorders or disabilities and numerous stressful life events among students (≥ 8 events) were correlated with higher overall GHQ-28 scores (B = 15.232, *p < .005*).

**Conclusion:**

The high frequency of clinical symptoms of mental distress among postgraduate university nursing students and their correlation with stressful life events highlights the need for university counselling services to provide psychological support strategies to students.

## Background

Depression and anxiety are among the most prevalent health issues worldwide, accounting for 30% of the non-terminal and non-fatal disease burden worldwide [1, 2]. According to the World Health Organization (WHO), millions of people are affected by some form of mental illness (approximately 416 million in 1990 to 615 million in 2013) [2], studies estimating the prevalence to be as high as 13–30% for depression and 18–31% for anxiety [3, 4].

Stressful life events are defined as independent measurable conditions, such as financial difficulties, social relationships, family and personal controversies, educational concerns, and health-related stressors, all of which may have a detrimental effect on mental health by increasing the risk of depression and anxiety [5–7]. Moreover, individuals who experience stressful circumstances are more likely to experience psychological issues [8, 9].

Stressful life events, either negative (e.g. death of parents or loved ones) or positive (e.g. getting married, finding a job), can heavily impact students’ life status and subsequently their physical and mental health [9, 10].

Master students’ issues are a particularly important period for mental health policy formulation as 75% of mental illnesses appear in the second decade of life [11]. However, mental health problems among university populations are still not well understood.

According to previous studies, graduate students are more than six times more likely to experience depression and anxiety than the general population, even though research has shown that university student populations around the world are changing and more closely resemble the general population in terms of risk factors and rates of psychopathology [12, 13].

Literature review among post university students, supported [13–15] that post graduate university students (study at Master or other programs) experience high levels of mental health distress syndrome (anxiety, stress and depression) compare with those students study in undergraduate university programs. The results pointed out that post graduate students schoolwork, money, graduate/teaching assistantships, career planning, and family concerns as the most common stressors [16].

Post graduate students and especially in health sciences, have significant rates of mental health distress (stress, anxiety and depression), which affect their mental health status [17], with negative effect on their personal, academic and social wellbeing. According to a Saudi Arabian study [18], a large percentage of graduate students studying in health sciences fields, usually, they experience mental health distress syndrome (stress, anxiety and depression) soon after commencing their course of study. More specifically, almost one out of 5 students (17%) presented moderate-to-severe depressive symptoms [18].

There is a consensus that nursing is a highly stressful profession, with nursing students, along with other healthcare professionals, being more prone to higher levels of anxiety, stress, and depression than students in other fields [19].

Nursing post graduate programs enable nursing students to improve their academic growth and are crucial for the nursing profession. Kaur et al. [20], assessed the levels of depression, anxiety, and stress among postgraduate nursing students and reported high levels of depression, anxiety, and stress during their postgraduate study [20].

Graduate nursing students face a number of difficulties, such as juggling their obligations to their families, jobs, and studies [21]. They so struggle to balancing their time well at home, at work, and academic achievement. Graduate students seek to fulfill their responsibilities for the family, which usually include housework and parenting, and frequently feel guilty when they are not able to do so well [22]. Additionally, it has been noted that nursing graduate students have challenges at work, including a lack of understanding from coworkers and scheduling issues [23].

Due to the difficulties outlined above, nursing graduate students feel stress, which tends to get worse as the program progresses [22, 24, 25].

Similar to above, Pezaro et al. [26], argued that midwives experience psychological distress due to work-related organizational and professional factors [26]. In the midwifery industry, it has been noted that there are toxic workplace environments, a lack of staff support, bullying, burnout, callous behaviors, compassion fatigue, and significant staff turnover rates [27, 28].

Furthermore, considering that many of these people are either current healthcare professionals or will become healthcare professionals in the near future, we must ensure that this workforce is capable of safe and practice. The presence of mental health problems among healthcare staff threatens their competency and may also lead to risks for patients [29]. Therefore, data on the association between mental health status and the number and severity of stressful life events may offer valuable information that can support relevant interventions for stress management and resilience building in vulnerable populations.

The primary aims of the present study are to (a) examine mental health status and subsequent predictors of clinically significant mental distress symptoms, (b) estimate the number and severity of stressful life events, and (c) explore the associations between mental health status, stressful life events (number and severity) and sociodemographic characteristics.

## Methods

### Design and study population

This descriptive, cross-sectional, correlational study was conducted at the Department of Nursing, Cyprus University of Technology (CUT). The study population consisted of postgraduate metal health nursing and midwifery nursing students. A convenience sample of nineteen (N = 90 out of N = 92) Master of Science (MSc) programs students (Mental health nursing and Midwifery students) studying at our university (CUT) participated in the present study. It is important to mention that, during our survey our university offers only the above postgraduates’ programs. As results the authors decided to involve all the students in this study. Specifically, the sample at the present study involved was collected from the three (3) masters programs in nursing. At the moment of collection of data were running two (2) mental health in nursing programmers and one (1) in nursing and midwifery program. A convenience sample of nineteen (N = 90 out of N = 92) students (Mental health nursing and nursing and midwifery) studying at our university (CUT) participated in the present study. It is important to mention that, during our survey, our university offers only the above postgraduates’ programs. As results the authors decided to involve all the master nursing students in this study.’

This study was approved by the Cyprus National Bioethics Committee [Ref. No: 2010.01.38]. All participants provided consent after being informed of the study’s goals and data-gathering methods. All active postgraduate students (N = 92) from the above programs were eligible to participate, regardless of their gender, age, or nationality. Students who were undergraduate or doctoral candidates were excluded. The final sample comprised 90 postgraduate nursing students (response rate: 97.8%).

### Instruments

The questionnaires included the Life Events Scale for Students (LESS), the General Health Questionnaire-28 (GHQ-28) a sociodemographic questionnaire and an educational leaflet outlining the study goals and procedures.

#### The sociodemographic questionnaire

A questionnaire was created for the purpose of the current study, the sociodemographic and other characteristics of the sample (such as the academic profile) were evaluated. The sociodemographic questionnaire design for the present study included questions (23 Items) pertaining to individual characterises (gender, age, place of residence, family status, and employment status), personal habits - substance abuse-related behaviour (alcohol consumption, smoking habit, use of drugs) parental status (parental marital status, loss of parent(s), parent’s level of education, parents’ employment status), academic status (level of satisfaction with the program of study, quality of the education system, learning difficulties ), social life characteristics (frequency of spending time with friends), questions concerning satisfaction with relationships (e.g. with parents and with friends) and final, health status (physical and mental health self-assessment during last month and chronic physical disorder or disability).

#### The GHQ-28

The Greek version (adapted by Garyfallos et al. [30]) of the General Health Questionnaire-28 was used to measure mental health status.

The GHQ-28 is widely used to screen individuals with minor mental disorders in the general population [31]. It includes 28 items rated on a 4-point Likert scale ranging from 0 = never to 3 = more than usual corresponding to four subscales: general health (somatic) symptoms (seven items), anxiety/insomnia symptoms (seven items), personal/social functioning (seven items), and depressive symptoms/suicidality (seven items). The total score on the GHQ-28 ranged from 0 to 84, whereas the scores on each subscale ranged from 0 to 21. Higher GHQ-28 scores correspond to higher levels of distress.

The GHQ-28 had satisfactory reliability (Cronbach’s alpha coefficients between 0.78 and 0.95) when tested in a student population [32]. For this study, Cronbach’s alpha was 0.90. A cut-off score of ≥ 24 has been suggested as representative of psychological disturbances in previous studies [33]. Thus, a cut-off score of ≥ 24 was classified as having notable mental health problems in the present study.

#### The life events Scale for students (LESS)

The Life Events Scale for Students (LESS) is a 36-item checklist created specifically for university students developed by Linden et al. [34] as a tool to identify high-risk student populations by predicting the probability of disease and illness and linking life events with psychological and health problems [34]. Participants were required to indicate whether they had encountered the described events, which were assigned weights based on how stressful they were thought to be within the previous 12 months [35]. The results were determined by adding the total weights of the items. Over a period of six months, the test-retest reliability of the individual items was 0.61 [35].

Some evidence suggests that life event weightings are cross-culturally generalizable [35, 36]. However, the possibility that certain events will be perceived differently by students based on their cultural backgrounds is relatively high. Therefore, in our previous pilot study, the scale was adapted to the Cypriot population [37].

### Data collection

The questionnaire was distributed to students in lecture theatres, and written informed consent was a prerequisite for their participation in the study. Participation in the study was anonymous and voluntary, and confidentiality was guaranteed. To protect their anonymity, students were instructed to not provide identifiers in the questionnaire. Additionally, those who wished to abstain from participating could do so by not answering the questionnaire. After being anonymously sealed in envelopes, the completed questionnaire packs were returned to the collection box. The research team ensured that the data collection procedure did not coincide with midterm and final examinations or any other potential studies related to stressful situations, such as clinical placements or internships, as previous studies have documented that stress levels increase in such conditions.

### Data analysis

Descriptive statistics for means and frequencies, t-tests, and one-way analysis of variance (ANOVA) were used to compare the GHQ-28 subscales and total mean scores between the different sociodemographic groups. Before statistical analysis of the data, a test was performed to check for normal distribution, where the Kolmogorov-Smirnov test was not statistically significant (p = .91), so we accepted the null hypothesis. Concerning the LESS, in both the number and severity groups, a chi-square test was conducted to compare the proportion of students with a total GHQ score above the recommended cut-off point with LESS scale scores. For this study, a cut-off value of 24 was used.

Before and after adjusting for potential confounders, logistic regression was used to assess the odds ratio and 95% confidence intervals of clinical mental distress symptoms (measured by the GHQ-28 ≥ 24) using demographic and other characteristics. Linear regression was used to estimate the effect of stressful events in relation to demographic variables on the dependent variable, GHQ-28 total score. The first quartile was further split into two separate categories from 0 to 49 and 50–150, the second quartile consisted of participants who scored from 150 to 241, the third quartile from 242 to 349, and the last quartile comprised the category with the highest score of the LESS score (i.e. 350–776). The number of stressful events was categorised using the same strategy. Statistical Package for Social Sciences Software (SPSS version 20) was used to analyse the data. For all statistical tests, *p* ≤ .05 were regarded as statistically significant.

## Results

### Sociodemographic characteristics of the sample

The final sample consisted of 90 postgraduate students (response rate: 97.8%) from the Cyprus University of Technology (CUT), Nursing Department, specifically students specialising in MSc Mental Health Nursing and Midwifery, of whom 47.8% were male (n = 43) and 52.2% (n = 47) were female. Most participants were between 22 and 26 years of age (n = 72, 80%), most were single (n = 55, 61.1%), approximately 64.4% (n = 58) lived in urban areas, and 56.7% (n = 51) were employed and the vast majority of the participants’ parents were married (n = 69, 76.7%). In terms of health status, just 8.9% (n = 8) of participants had a chronic physical disability, while most self-assessed both their physical (n = 65, 72.2%) and mental health (n = 56, 62.2%) as excellent or very good. Moreover, half of the participants were satisfied with the quality of the education system (n = 46, 50.1%). Tables 1, 2 and 3 present the participants’ sociodemographic characteristics.

**Table 1:** The mean score in GHQ-28 of total score and subscales by classification of participants in terms of basic socio-demographic characteristics

Sociodemographic Characteristics	N	(%)	GHQscore, mean ± SD	P	Somatic symptoms 1–7(items)	P	Αnxiety / insomnia 8–14 (items)	P	Social dysfunction 15–21 (items)	P	Severe depression 22–28 (items)	P
**Gender**				0.964		0.121		0.560		0.091		**0.034**
Male	43	47.8	22.95 ± 11.39		6–16 ± 3.23		7.21 ± 4.86		6.58 ± 3.24		3.0 ± 3.69	
female	47	52.2	19.74 ± 10.92		19.74 ± 3.57		6.62 ± 4.73		6.51 ± 2.72		1.60 ± 2.42	
**Total**	90	100%	21.0 ± 11.20		5.57 ± 3.48		6.90 ± 4.71		6.545 ± 2.96		2.27 ± 3.15	
**Age**				0.335		0.411		0.146		0.292		**0.008**
*22–26*	72	80	21.23 ± 10.55		5.56 ± 3.42		6.90 ± 4.67		6.62 ± 2.93		2.14 ± 2.73	
*27–30*	15	16.7	23.13 ± 14.28		5.75 ± 3.58		7.69 ± 5.47		6.56 ± 3.16		3.13 ± 4.77	
*31–36*	3	3.3	12.67 ± 3.78		4.67 ± 7.68		2.67 ± 1.15		4.67 ± 0.57		0.67 ± 3.15	
**Place of residence**				0.992		0.795		0.795		0.225		0.871
*Urban area*	58	64.4	21.55 ± 11.16		5.65 ± 3.45		7 ± 4.58		6.47 ± 2.75		2.42 ± 3.52	
*Sub-urban area*	23	25.6	21.50 ± 11.33		4.48 ± 2.29		5.88 ± 3.72		8.25 ± 2.60		2.50 ± 2.39	
*Rural* areas	9	10	21.19 ± 8.75		5.86 ± 3.97		7.19 ± 5.60		6.14 ± 3.61		2 ± 2.72	
**Family status**				0.522		0.055		0.170		0.387		0.378
*Single*	55	61.1	21.62 **±** 10.89		5.56 ± 3.58		6.69 ± 4.75		6.87 ± 2.93		2.49 ± 3.73	
*Married*	21	23.3	22.43 **±** 12.73		6.14 ± 3.87		8.00 ± 5.29		6.52 ± 3.23		1.76 ± 2.93	
*living with partner*	14	15.6	18.21 **±** 10.19		4.71 ± 2.30		6.07 ± 4.08		5.29 ± 2.52		2.14 ± 2.67	
**Employment**				**0.014**		0.521		0.688		**0.042**		0.399
*Yes*	51	56.7	21.3 ± 12.34		3.74 ± 3.66		6.47 ± 3.51		5.89 ± 3.08		1.79 ± 2.20	
*No*	39	43.3	19.89 ± 8.31		6.47 ± 3.51		6.86 ± 5.32		6.98 ± 2.89		2.29 ± 3.24	
**Smoking habit**				0.832		0.963		0.245		0.697		0.743
*No*	48	53.3	21.04 ± 10.39		2.27 ± 0.96		2.22 ± 0.91		2.22 ± 0.77		1.23 ± 0.56	
*Yes*	42	46.7	21.55 ± 12.18		2.26 ± 0.85		2.0 ± 0.81		2.16 ± 0.72		1.27 ± 0.59	
**Alcohol consumption**				**0.001**		**0.001**		**0.008**		**0.011**		0.143
*No*	40	44.5	25.60 ± 11.92		6.95 ± 3.58		8.58 ± 5.52		7.33 ± 2.92		2.75 ± 3.85	
*Rare/occasionally*	37	41.1	16.16 ± 9.28		3.97 ± 2.86		5.27 ± 3.76		5.43 ± 2.84		1.88 ± 2.46	
*Often/very often/daily*	13	14.4	22.54 ± 8.06		5.85 ± 3.07		6.38 ± 3.12		7.31 ± 2.62		2.27 ± 3.15	
**Drugs addiction**				0.343		0.166		0.122		0.578		0.532
*No drugs use*	81	90	20.85 ± 11.07		2.2 ± 0.86		2.14 ± 0.90		2.18 ± 0.76		1.24 ± 0.55	
*Rare/occasionally*	9	10	25.11 ± 12.272		2.66 ± 1.22		1.85 ± 0.37		2.33 ± 0.70		1.27 ± 0.74	

*The bold are statistically significant

**Table 2:** The mean score in GHQ-28 of total score and subscales by classification of participants in terms of basic parental status

Sociodemographic Characteristics	N	(%)	GHQscore, mean ± SD	P	Somatic symptoms 1–7(items)	P	Αnxiety / insomnia 8–14 (items)	P	Social dysfunction 15–21 (items)	P	Severe depression 22–28 (items)	P
**Parental marital status**				**0.023**		0.696		0.139		0.301		**0.017**
*Married*	69	76.7	20.22 ± 10.71		5.02 ± 3.36		6.35 ± 4.27		6.78 ± 2.61		2.08 ± 2.82	
*divorce*	14	15.5	29.92 ± 10.62		8.00 ± 2.59		10.33 ± 4.51		7.00 ± 3.17		4.58 ± 4.56	
*widow*	7	7.8	20.71 ± 13.89		5.00 ± 2.88		8.29 ± 7.20		5.57 ± 3.30		1.88 ± 3.23	
**Father’s educational level**				0.434		0.259		0.069		0.592		0.483
*Primary*	30	33.3	21.97 ± 10.12		6.23 ± 3.73		7.03 ± 4.26		7.0 ± 3.29		1.70 ± 2.35	
*Secondary*	48	53.4	20.04 ± 11.92		5.0 ± 3.27		6.13 ± 4.76		6.33 ± 2.88		2.58 ± 3.69	
*University*	12	13.3	24.50 ± 10.90		6.17 ± 3.56		9.67 ± 5.39		6.25 ± 2.52		2.42 ± 2.53	
**Mother’s educational levell**				0.704		0.151		0.847		0.998		0.213
*Primary*	27	30	20.78 ± 11.79		5.93 ± 4.03		6.93 ± 5.17		6.56 ± 2.95		1.37 ± 2.259	
*Secondary*	51	56.7	22.04 ± 10.97		5.80 ± 3.32		7.06 ± 4.47		6.53 ± 3.02		2.65 ± 3.20	
*University*	12	13.3	19.17 ± 11.48		3.75 ± 2.26		6.17 ± 5.49		6.59 ± 3.02		2.67 ± 3.15	
**Father’s employment status**				0.010		0.248		0.070		0.208		0.112
*Unemployed*	66	73.3	23.77 ± 11.68		2.34 ± 0.93		2.25 ± 0.82		2.27 ± 0.79		1.30 ± 0.63	
*Employed*	24	26.7	15.88 ± 928		2.0 ± 0.826		1.82 ± 0.95		2.05 ± 0.55		1.11 ± 0.33	
**Mother’s employment status**				0.331		0.208		0.265		0.044		0.027
*Unemployed*	39	43.3	22.67 ± 11.14		2.41 ± 0.96		2.25 ± 0.99		2.38 ± 0.74		1.10 ± 0.39	
*Employed*	51	56.7	20.32 ± 11.33		2.16 ± 0.86		2.02 ± 0.76		2.06 ± 0.73		1.36 ± 0.66	
**Loss of parent(s)**				0.871		0.803		0.872		0.625		0.526
Yes	10	11.2	20.7 ± 11.7		5.54 ± 3.55		6.86 ± 4.61		6.61 ± 2.88		2.34 ± 3.20	
No	80	88.8	21.2 ± 11.2		5.80 ± 0.30		7.20 ± 6.25		6 ± 3.71		1.70 ± 2.86	

*The bold are statistically significant

**Table 3:** The mean score in GHQ-28 of total score and subscales by classification of participants in terms of self-reported academic, social life and health status

Sociodemographic Characteristics	N	(%)	GHQscore, mean ± SD	P	Somatic symptoms 1–7(items)	P	Αnxiety / insomnia 8–14 (items)	P	Social dysfunction 15–21 (items)	P	Severe depression 22–28 (items)	P
**Level of satisfaction with quality of the education system**				**0.003**		0.156		0.171		0.087		**0.004**
No/ Low	44	49.9	24.82 ± 11.68		2.4 ± 0.92		2.27 ± 0.87		2.34 ± 0.77		1.43 ± 0.70	
Hight/ Very high	46	50.1	17.93 ± 9.78		2.1 ± 0.89		2.0 ± 0.87		2.0 ± 0.71		1.08 ± 0.35	
**Level of satisfaction with program/course of study**				0.243		0.449		0.132		0.066		**0.022**
No/ Low	34	37.7	23.65 ± 12.28		2.24 ± 0.89		2.27 ± 0.87		2.35 ± 0.78		1.43 ± 0.68	
Hight/ Very high	56	62.3	20.38 ± 10.75		2.09 ± 0.86		2.0 ± 0.87		2.0 ± 0.65		1.04 ± 0.21	
**Learning difficulties**				**0.012**		0.260		**0.023**		**0.004**		0.063
No	68	75.6	19.63 ± 10.37		5.40 ± 3.43		6.23 ± 4.51		6.06 ± 2.88		1.94 ± 3.05	
Yes	22	24.4	27.27 ± 12.05		6.41 ± 3.62		9.18 ± 5.17		8.18 ± 2.71		3.50 ± 3.37	
**Satisfaction with relationship with friends**				0.172		0.786		**0.041**		0.602		0.229
No/ Low	14	15.6	25.57		5.71 ± 2.99		9.43 ± 4.65		7 ± 3.66		3.43 ± 3.86	
Hight/ Very high	76	84.4	20.44		5.47 ± 3.55		6.44 ± 2,86		6.46 ± 2.86		2.08 ± 3.01	
**Satisfaction with relationship with parents**				0.290		0.386		0.181		0.926		0.367
No/ Low	14	15.6	24.64 ± 12.97		2.27 ± 0.46		2.45 ± 0.68		2.18 ± 0.87		1.09 ± 0.30	
Hight/ Very high	76	84.4	20.61 ± 10.98		2.11 ± 0.89		2.07 ± 0.89		2.15 ± 0.71		1.24 ± 0.55	
**Frequency of spending time with my friends**				**0.003**		**0.008**		**0.001**		0.200		0.444
Low	34	37.8	25.9 ± 10.99		6.79 ± 3.05		9.24 ± 4.52		7.21 ± 3.63		2.68 ± 2.32	
Hight	39	43.3	19.63 ± 11.56		5.11 ± 3.76		5.89 ± 4.90		6.32 ± 2.66		2.32 ± 3.68	
Very high	17	18.9	15.53 ± 7.19		3.82 ± 2.58		4.53 ± 3.04		5.71 ± 1.86		1.47 ± 2.06	
**Physical health self-assessment during last month**				**< 0.01**		**0.011**		**0.026**		**0.017**		**0.024**
Excellent/very good	65	72.2	18.61 ± 9.97		6.16 ± 4.46		6.06 ± 2.64		6.19 ± 2.31		2.05 ± 2.90	
Good	17	18.9	30.41 ± 11.37		10 ± 5.0		8.24 ± 3.45		8.30 ± 3.45		4.17 ± 3.70	
Poor/very poor	8	8.9	22.88 ± 11.51		6.38 ± 4.98		6.75 ± 2.98		8.30 ± 2.80		2.29 ± 3.16	
**Mental health self-assessment during last month**				**< 0.001**		**0.001**		**< 0.001**		**0.024**		**0.002**
Excellent/very good	56	62.2	16.64 ± 7.83		4.72 ± 2.70		4.36 ± 3.15		5.75 ± 2.61		1.41 ± 2.0	
Good	25	27.7	26.76 ± 11.22		6.64 ± 3.08		9.25 ± 5.14		7.20 ± 2.83		3.68 ± 4.27	
Poor/very poor	9	10.1	36.25 ± 12.03		10.3 ± 1.15		13 ± 4.8		8.63 ± 4.59		4.13 ± 3.70	
**Chronic physical disorder or disability**				0.241		0.137		0.379		0.326		**0.04**
No	82	91.1	21.13 ± 10.39		5.06 ± 3.14		7.30 ± 4.60		6.64 ± 2.62		2.13 ± 2.77	
Yes	8	8.9	28 ± 14.74		6.88 ± 3.09		8.88 ± 4.97		7.75 ± 4.46		4.50 ± 4.30	

*The bold are statistically significant

### GHQ-28 total scores/subscale scores

The minimum and maximum GHQ-28 total scores were 1 and 56, respectively (possible range: 0–84). In terms of subscale scores, the minimum and maximum scores were as follows (possible range: 0–21): somatic symptoms: 0–14 (, anxiety/insomnia symptoms: 0–19, personal/social functioning: 0–16, and depressive symptoms/suicidality: 0–13 (range: 0–21 for each subscale). Mean values for all variables assessed are presented in Tables 1, 2 and 3.

### Associations between GHQ-28 total score/ subscales score and sociodemographic characteristics

The mean and standard deviation of the GHQ-28 total score were 21.0 ± 11.2. There were no statistically significant differences between the GHQ-28 total score and gender, age, place of residence, or family status. In contrast, employed participants reported higher GHQ-28 scores compared to unemployed individuals (*p* = .014). Additionally, in terms of substance use, statistically significant differences were observed in only alcohol use, with participants who had never consumed alcohol reporting higher scores in GHQ-28 total scores [25.60 (± 11.92), *p* = .001] (Table 1).

With regard, to students’ parental status, those whose parents were divorced reported statistically significantly higher GHQ-28 scores compared to those whose parents were married or widowed, (*p* = .023). There are no statistically significant differences in the relation between parental employment status and the total score of GHQ-28(Table 2).

Regarding the GHQ-28 subscales, alcohol consumption appeared to make statistical differences on three of the four subscales (except the severe depression subscale) with participants who did not consume alcohol scoring higher than those who did Rare/occasionally, Often/very often or daily(*p* < .05). There were no statistically significant sociodemographic differences in the somatic symptoms and anxiety/insomnia subscales, whereas, for the social dysfunction subscale, statistically significant differences were noted only in relation to employment status. Specifically, unemployed participants reported higher scores than employed participants (*p* = .014). Concerning, depressive symptoms/suicidality, males reported higher scores compared to females (*p* =. 034), those aged 27–30 years reported higher scores compared to those aged 22–26 and 31–36 years *(p* = .008), and those whose parents were divorced compared to those whose parents were married or widowed ( *p* = .017). Tables 1 and 2 presents these data.

### Associations of GHQ-28 total score/subscale scores and academic, social life, and health status

With regard to academic characteristics, statistically significant differences in GHQ-28 total scores were noted by learning difficulties (*p* = .012) and level of satisfaction with the quality of the education system, with participants who were low/not satisfied with the education system having higher scores in relation to participants reporting having high/very high satisfaction levels (*p* < .05). Additionally, higher scores on the GHQ-28 were observed among students who spend less time with their friends [25.9 (± 10.99), *p* < .005].

Furthermore, in terms of health status, mental health during the last month had significant differences between participants who self-assessed their mental health as poor or very poor compared to those who self-assessed their mental health as very good or excellent (*p* <. 001). Furthermore, the physical health self-assessment showed significant differences between those who described their physical health as good in relation to those who self-assessed it as poor/very poor [22.8 (± 11.51), (*p* <. 01)] or very good/ excellent [18.61 (± 9.97) (*p* <. 01)]. In contrast, the chronic physical disorder and disability groups showed no statistically significant differences in mean values.

Concerning the GHQ-28 subscales, higher scores of severe depression were observed in participants who had a chronic physical disorder or disability (*p* < .05).

Additionally, higher mean value was noted among those who self-assessed their physical and mental health as poor or very poor with statistically significant differences with a *p* value < 0.05, except for the severe symptoms subscale where participants who self-rated their physical health as good scored higher (*p* < .005).

Participants with learning difficulties appeared to score higher on the subscale of anxiety/insomnia and social dysfunction (*p* < .05). On the other hand, the social life had noted high score for anxiety/insomnia and somatic symptoms. Specifically, the satisfaction with relationships with friends had noted high score for anxiety/insomnia *(p* < .05) and the frequency of time spend with their friends on both subscales [*p* < .005, (somatic symptoms), *p* < .05 (anxiety/insomnia)], while the level of satisfaction with the education system and program/course of the study appeared to have a greater effect on severe depressive symptoms.

### Number and severity of reported stressful life events and correlation with total GHQ-28 score

Table 4 presents the number of the reported stressful life events. The severity ranking of stressful life events, as shown in Table 4, was reported in a previous study conducted by Sokratous et al. [37] using a pilot sample of students. Approximately 57.8% of the participants reported vacations alone or with friends and beginning an under.

**Table 4:** Number and ranking of LCU reported stressful life events on the LESS scale in Cypriot post-graduate university students and corelation with total score of GHQ-28

Less scale for university students	Rank of the severity+	N	%	kendall’s tau Correlation
1. Death of parent	100	4	4.4	0.067
2. Death of your best friend or very close friend	91	17	18.9	0.015
3. Major car accident (car wrecked, people injured)	83	3	3.3	0.012
4. Major personal injury or illness	82	10	11.1	0.343**
5. Getting kicked out of college	76	0	0	-
6. Major and/or chronic financial problems	76	19	21.1	0.188*
7. Break-up of parent’s marriage/divorce	72	2	2.2	0.066
8. Seriously thinking about dropping college problems	72	9	10	0.450**
9. Failing in one course	71	6	6.7	0.109
10. Losing a part-time job	69	10	11.1	0.053
11. Parent losing his/her job	68	7	7.8	0.120
12. Pregnancy (either yourself or being the father)	64	10	11.1	-0.157
13.Failing in a number of course	64	13	14.4	0.133
14. Sex difficulties with boy/girlfriend	64	6	6.7	0.261*
15.Jail term (self)	62	1	1.1	0.012
16. Breaking up/ loosing contact/with a close friend	62	18	20	0.072
17.Breaking up with boy/girlfriend	62	8	8.9	-0.068
18.Major argument with boy/girlfriend	60	11	12.2	0.056
19.Major change of health status in a close family member	57	27	30	0.039
20.Minor financial probleml	57	37	41.1	0.089
21. Getting an unjustified low mark in a test	54	8	8.1	0.171
22. Major argument with parents	53	15	16.7	0.059
23. Moving away from home	52	12	13.1	-0.444
24. Moving out of town with parents	51	2	2.2	0.086
25. Change of job	50	13	14.4	-0.048
26. Seeking psychological or psychiatric consultation	49	6	6.7	0.169
27. Switch in a program within the same college or university	47	6	6.7	0.017
28. Establishing a new steady relationship with a partner	39	6	6.7	-0.041
29. Minor car accident	38	17	17.9	-0.093
30. Getting your own car	33	14	15.6	-0.100
31. Finding a part-time job	33	11	12.2	-0.079
32. Beginning an undergraduate program in the university	31	52	57.8	
33. Minor violation of the law (e.g. speeding ticket)	27	21	23.3	-0.040
34. Family getting together	22	39	43.3	-0.170*
35. Vacation with parents	15	22	22.4	-0.199*
36. Vacation alone/with friends	14	52	57.8	-0.253**

+ Severity ranking given in a previous study by university students in Cyprus. All significant correlations are in bold, *p < .05, **p < .001. The scale assesses the severity of the experienced stress following these events by using Life Change Units (LCU). Each LCU score, assigned to each stressful life event, which might be a major or a minor life situation, positive or negative, reflects the amount of readjustment an individual has to make in order to regain homeostasis

Graduate program at the university as the most stressful event. The less stressful reported events were jail terms (1.1%) and the breakup of parents’ marriage/divorce (2.2%). Generally speaking, participants rated minor financial problems, family get- togethers, vacations with parents, minor violations of the law, and major changes in the health status of a close family friend as the top 10 stressful life events. In the bottom 10, establishing a new steady relationship with a partner, sexual difficulties with a partner, switching programs within the same university, seeking psychological consultation, failing one course, moving out of town with parents, death of a parent, a jail term, parents’ divorce, and major car accidents were reported. Moreover, most participants reported experiencing 3–6 stressful life events.

Kendall’s Tau correlation test was used to determine the correlation between stressful events and the total GHQ-28 score. The results of the correlation analysis showed that the stressful life events showing the strongest positive correlation were ‘Seriously thinking about dropping college problems’ and ‘Major personal injury or illness’ with Kendall’s tau correlations of 0.450 and 0.343, respectively, *p <. 001*. On the other hand, the strongest statistically significant negative correlation was ‘Vacation alone/with friends’ with a Kendall’s Tau correlation of -0.253, *p* <. 05.

### Associations between the GHQ-28 (total score ≥ 24) and stressful life events

Of the 90 students, 33 (36.7%) reported experiencing clinically significant mental distress. Concerning the associations between mental health status (GHQ-28 total score ≥ 24) and stressful life events, no statistically significant difference between the two groups was observed either in the total score on the LESS or in the number of events on the LESS and clinical symptoms of mental distress (see Table 5). However, Table 5 presents the prevalence of clinical mental distress symptoms in terms of the number of reported life events and the overall LESS scores associated with these events. Among participants who did not report any stressful life events in the last 12 months, only one (1.1%) showed mental distress symptoms (GHQ-28 total score ≥ 24). The prevalence of mental distress symptoms was zero among those with scores in the range of 0–49, but gradually increased at higher LESS scores, reaching 52.6% among the quartile of participants in the highest score category.

**Table 5:** Prevalence of clinical mental distress (GHQ-28 ≥ 24) by classification of participants in terms of the number of stressful life events and total score on the LESS scale

Life Events Scale for Students (LESS) (N = 90)	Total	Non clinical mental distress		Prevalence of clinical mental distress		X^2^	DF	P value
		N	%	N	%			
**Number of events in LESS**	0–3	16	64	10	36	0.106	2	0.949
	4–7	29	67.4	14	32.6			
	8–14	12	57.1	9	42.9			
						6.251	4	0.181
**Total Score in LESS**	0–49	5	100	0	0			
	50–149	13	59.1	9	40.9			
	150–241	13	65	7	35			
	242–350	17	70.8	7	29.2			
	351–767	9	47.4	10	52.6			
**Total**		57	63.3	33	36.7			

### Associations between clinical mental health distress (GHQ-28 ≥ 24) by sociodemographic characteristics and self-assessment of participants’ health

When multiple logistic regression analysis was performed with clinical mental distress (GHQ-28 ≥ 24) as the dependent variable and sociodemographic characteristics and self-assessment of participants’ health as the independent variables (Table 6), it was observed that males had 5.5 times greater odds of having a higher GHQ-28 score (> 24) than females. Moreover, participants whose parents were divorced were more likely to have clinical symptoms of mental distress than those whose parents were still married. Participants who reported no or low satisfaction with their relationships with friends were 33.4 times more likely to have clinical symptoms of mental distress than those who reported low satisfaction with their friendships. In contrast, participants who declared that they were very satisfied with their parents had significantly lower odds of experiencing clinical symptoms of mental distress than those who had low satisfaction with their parents. Furthermore, those who reported spending considerable time with friends had lower odds of experiencing clinical symptoms of mental distress. Finally, those who self-assessed their mental health as poor or very poor in the past month had 9.72 times greater odds of experiencing clinical symptoms of mental distress than those who self-assessed their mental health as excellent or very good.

**Table 6:** Adjusted odds ratios (and 95% CI) of clinical mental distress (GH28 ≥ 24) by sociodemographic characteristics and self-assessment of participants’ health as estimated in multivariable backward stepwise logistic regression analysis

GHQ-28 (≥ 22)	B	S.E	Wald	DF	Adjusted		P value
					OR	CI(95%)	
Gender							
Female					1		----------
Male	1.705	0.669	6.500	1	5.50	1.48–20.41	**0.011**
**Age**							
22–27					1		----------
28–36	-1.260	1.089	1.340	1	4.83	1.24–18.86	0.247
**Parental status**							
*Married*					1		----------
*divorce*	3.645	1.465	6.186	1	38.2	2.16- 676.65	**0.013**
**Father’s employment status**							
*Unemployed*					1		----------
*Employed*	-1.599	0.907	3.109	1	0.20	(0.03–1.62)	0.708
**Learning difficulties**							
No					1		----------
Yes	1.049	0.772	1.847	1	2.85	0.62–12.96	0.174
**Alcohol consumption**							
No							
Very often/occasionally	-0.989	0.753	1.725	1	0.37	0.08–1.62	0.189
**Chronic physical disorder or disability**							
No					1		----------
Yes	-0.551	0.872	0.399	1	0.57	0.10–3.18	0.527
**Loss of parentNOs)**							
No					1		----------
Yes	-0.790	1.154	0.469	1	0.45	0.04–4.35	0.493
**Level of satisfaction with quality of the education system**							
No/ Low					1		----------
Hight/ Very high	-1.217	1.075	1.282	1	12.17	1.30-113.71	0.258
**Level of satisfaction with program/course of study**							
No/ Low					1		----------
Hight/ Very high	-1.184	0.620	3.644	1	0.30	0.09–1.10	0.056
**Satisfaction with relationship with friends**							
No/ Low					1		----------
Hight/ Very high	3.510	1.197	8.600	1	33.46	3.07-130.97	**0.002**
**Satisfaction with relationship with parents**							
No/ Low					1		----------
Hight/ Very high	-0.960	0.872	1.213	1	0.38	0.69–2.11	0.029
**Frequency of spending time with my friends**							
Low					1		----------
Hight/Very hight	-2.072	0.689	9.039	1	0.126	0.03–0.486	**0.003**
**Mental health self-assessment during last month**							
Excellent/very good					1		----------
Poor/very poor	2.275	0.663	11.781	1	9.72	2.65–35.65	**0.001**
**Physical health self-assessment during last month**							
Excellent/very good					1		----------
Poor/very poor	0.153	0.773	0.039	1	1.11	0.25–5.30	0.843

^+^Variables included in the first stage: age, gender, loss of parent(s), Learning difficulties, Chronic physical disorder or disability, Physical health self-assessment during last month, Mental health self-assessment during last month, Level of satisfaction with program/course of study, Satisfaction with relationship with friends, satisfaction with relationship with parents and frequency of spending time with friends

### Multiple linear regression analysis for total GHQ-28 score by sociodemographic, academic, individual characteristics, and self-assessments of participants’ health

Two models of multiple linear regression analyses (see Table 7) were performed, with the total GHQ-28 score as the dependent variable. In the first model, sociodemographic, academic, and individual characteristics and self-assessments of health were used as the independent variables for those with the highest total LESS score. Males had greater total average GHQ-28 scores than females. Moreover, the loss of parents was associated with total GHQ-28 scores in multiple analyses. Worse mental health self-assessments during the last month and worse levels of satisfaction with the program or course of study and less time they spent with friends were associated with higher GHQ-28 total scores in multiple analyses. Additionally, higher satisfaction with parental relationships and higher frequency of spending time with friends were independently associated with lower GHQ-28 total scores. These seven variables (gender, loss of parents, mental health self-assessments during the last month, levels of satisfaction with the program or course of study, and satisfaction with parental relationships and frequency of spending time with friends) predicted total LESS score and accounting for the 82.2% (adjusted R^2^ = 0.822, p < .001). In the second model, sociodemographic, academic, and individual characteristics and self-assessments of health were used as independent variables for those with the greatest number of stressful life events. It was found that males had a greater total GHQ-28 score than females. Moreover, chronic physical disorders and disabilities were associated with higher total GHQ-28 scores. Additionally, satisfaction with relationships with parents and the loss of parents were found to be associated with the total GHQ-28 scores in multiple models. In the second model, these four variables (gender, chronic physical disorders and disabilities, satisfaction with relationships with parents and the loss of parents) predicted the number of stressful events and accounted for 64.5% (adjusted R^2^ = 0.645) of the total variance.

**Table 7:** Multiple Linear regression analysis for total score of GHQ-28 by sociodemographic, academic, individual characteristics and self-assessment of participants’ health

**(a)**	**Unstandardized Coefficients**		**Standardized Coefficients**	**T**	**P value**
	**B**	**S. E**	**B**		
(Constant)	23.780	3.258		7.296	0.000
**Gender**	11.243	2.773	0.446	4.055	0.001
**Age**	5.145	6.418	0.144	0.802	0.438
**Learning difficulties**	1.173	3.659	0.059	0.474	0.646
**Loss of parent(s)**	-17.046	3.250	-0.582	-5.245	< 0.001
**Parental status**	2.236	3.766	0.900	0.594	0.566
**Chronic physical disorder or disability**	7.343	4.268	0.234	1.740	0.105
**Physical health self-assessment during last month**	-1.931	3.710	-0.777	-0.521	0.614
**Mental health self-assessment during last month**	10.798	2.673	0.428	4.039	0.011
**Level of satisfaction with program/course of study**	9.793	3.346	0.370	2.997	0.010
**Satisfaction with relationship with friends**	1.680	5.574	0.063	0.303	0.707
**Satisfaction with relationship with parents**	-7.343	2.968	-0.277	-2.474	0.027
**Frequency of spending time with my friends**	-12.197	3.305	-0.434	-3.690	0.002
**(b)**	**Unstandardized Coefficients**		**Standardized Coefficients**	**T**	**P value**
	**B**	**S. E**	**B**		
(Constant)	39.351	4.008		9.817	0.000
**Gender**	13.151	3.925	0.446	3.350	0.005
**Age**	10.552	7.103	0.268	1.486	0.161
**Parental status**	1.591	3.785	0.555	0.420	0.683
**Learning difficulties**	-0.783	3.343	-0.026	0.234	0.820
**Chronic physical disorder or disability**	15.232	4.483	0.433	3.398	0.004
**Physical health self-assessment during last month**	-5.077	4.548	-0.181	-1.116	0.290
**Mental health self-assessment during last month**	6.383	3.890	0.226	1.641	0.127
**Level of satisfaction with program/course of study**	0.964	5.109	0.034	0.189	0.856
**Satisfaction with relationship with friends**	-0.017	5.690	-0.001	-0.003	0.998
**Satisfaction with relationship with parents**	-12.142	3.476	-0.412	3.350	0.004
**Frequency of spending time with my friends**	-5.461	4.151	-0.194	-1.316	0.215
**Loss of parent(s)**	-18.292	3.834	-0.563	-4.771	< 0.001

(a)Backward stepwise multiple linear regression analysis with case with the highest* total score on the LESS scale and (b) the greatest** number of stressful life events using the GHQ-28 score as the outcome variable, and age, gender, loss of parent(s), Learning difficulties, Chronic physical disorder or disability, Physical health self-assessment during last month, Mental health self-assessment during last month, Level of satisfaction with program/course of study, Satisfaction with relationship with friends, satisfaction with relationship with parents and frequency of spending time with friends as independent variables

* According to the quartile of students, the highest score of LESS was ≥ 340, ** the quartile of students, the greatest number of stressful life events was ≥ 8

†(a)Adjusted R^2^ = 0.822 and (b) Adjusted R^2^ = 0.645

## Discussion

Researchers, more often focus their research study to undergraduate student population comparing with post graduate student population. The above creating a gap in the literature between these two populations. There is a huge amount of literature concerning mental health issues among undergraduate students. On the contrary, there is a need for more researches evidences of mental health issues on this specific population.

This study was the first to evaluate the mental health status and stressful life events of postgraduate nursing students in Cyprus and therefore add more research evidences in to the existing literature. There is an attempt to fulfill the gap in the literature between these special populations.

Furthermore, in the present study it was found a strong positive association between the prevalence of clinically significant mental health symptoms and stressful life events, both in terms of the reported number out of 36 events, as well as the total score as measured by the LESS, reflecting the severity associated with these events.

The study findings indicated that the prevalence of mental and clinical distress among postgraduate students was 36.7% (N = 33), which is consistent with those of previous studies. According to numerous studies, [13, 38–40] most postgraduate students worldwide face significant levels of stress and experience new patterns of mental health crises [13, 41, 42]. Research evidences supported that, 39% of post graduate students involved, experience signs of moderate-to-severe depression, which is six times higher than the general population [13].

An interesting finding of this study was the positive association between employment and higher GHQ-28 scores, indicating mental health difficulties among employed and unemployed students. It has been well documented that work-related stress can have a detrimental effect on mental and physical well-being [43].

The combination of academic studies and paid work has also been associated with detrimental impacts on students in several studies. Employed students find it difficult to balance the responsibilities related to paid work and commitment in relation to their studies [44]. Employment among full-time students impedes the progress of their academic achievement and limits time for studying [45]. Since work absorbs students’ time, the odds are against them successfully carrying out their academic obligations [46].

A contradictory finding of the present study was the positive association between higher total scores on the GHQ-28 and its subscales (somatic symptoms, anxiety/insomnia, and social dysfunction) in participants who never consume alcohol. Most of the existing literature reports an association between mental health problems, distress, and alcohol consumption in university and college student population [47–50].

A possible explanation for this discrepancy is that academic training in nursing, even at the pre-university level, is positively associated with less frequent drug and alcohol use [51]. However, it has also been suggested that a decrease in risky behaviours does not usually occur during the course, implying that nursing studies do not always follow good habits [52].

Moreover, the proportion of university students who use marijuana/hashish and alcohol continues to be much higher than that anticipated from educational programs on health, including nursing [53]. Further research with larger sample sizes is needed for a safer interpretation of this association.

Consistent with most published studies, statistically significant differences were reported in the higher GHQ-28 total scores between participants whose parents were divorced and those whose parents were still married. Geshica et al. [54] examined the association between parental marital status and psychological distress in college students and found that participants raised by married parents were more likely to experience lower psychological distress than those raised by divorced or widowed parents [54].

Interestingly, studies on the general population are not in agreement, with a number of studies suggesting that individuals with separated parents are more prone to encountering adverse mental health outcomes in adulthood, [55–57] and others indicating no association between those whose parents were divorced in childhood and those whose parents remained married [58].

Furthermore, a positive association has been reported among higher GHQ-28 scores, learning difficulties, and poor satisfaction with the education system. The highly structured nursing curriculum involves the need to be acquainted with and memorise knowledge and to blend, combine, and apply previously acquired knowledge from all learning domains related to nursing studies [59]. Studies have that the educational environment of medical sciences is perceived as stressful and negatively affects students’ educational performance and well-being [60]. Our results are consistent with those of a cross-sectional study of 710 pre-engineer students conducted by Bitew et al., [61] in which learning difficulties independently predicted increased depressive symptoms [61].

According to earlier surveys, students with higher levels of mental distress are more likely to experience negative consequences such as significantly impaired cognitive abilities, [62] poor academic performance, [63] learning disabilities, [64] higher risk of depression, [12] and anxiety disorders [65].

It is well known that mental distress can lead to learning difficulties and hinder academic educational achievement [66].

Concerning poor satisfaction with the educational system and the association with higher GHQ-28 scores, our results are consistent with a number of studies indicating that satisfaction with studies has a substantial impact on depression, anxiety, stress, and psychological well-being [67, 68]. Nerdrum et al. [69] studied psychological distress among nursing, physiotherapy, and occupational therapy students and found that nurses reported the least clarity in program structure, the strongest experience of excessive workload, and the lowest student climate quality [69]. A proper academic environment can help graduate students obtain scientific and clinical experience [69]. The ideal environment prepares students for their professional future and motivates their professional progress as well as physical, psychological, and social eudemonia. [67–69].

Self-reported health is generally considered a valid measure of health status and is widely used in survey research to estimate general health. In accordance with the above statement, we evaluated students’ self-assessments of their mental health during the past month. In line with the existing literature, participants who evaluated their health as poor or very poor were almost ten times more likely to present with clinically significant symptoms of mental distress (GHQ-28 > 24). Thus, the results of previous studies agree with our findings [37, 63, 64].

One of the most interesting findings of our study was the sex differences in depressive symptoms with males reporting higher scores on the depressive symptom’s subscale than females. Furthermore, when multiple regression analysis was performed, males had 5.5 times greater odds of presenting with clinically significant symptoms of mental health distress (GHQ-28 score > 24) than females.

Previous studies have reported a higher prevalence of depressive symptoms in females. The results of a number of studies are consistent with our findings, suggesting that male students present higher levels of depressive symptoms than their female counterparts [70–72].

Additionally, research has revealed that depression is one of the main risk factors for suicide attempts among nursing students [73]. Data from longitudinal studies have shown that if students fail to receive appropriate help, these symptoms persist for an extended period [74].

In our study, suicidality was significantly associated with the male sex. Consistent with our findings, in Halikopoulou et al.’s [75]. study on nursing students in Greece (even though no statistical association between gender and depressive symptoms was found), a higher prevalence of suicidal thoughts (without actually leading to suicide) was reported by male students than females [75].

Additionally, the current study found higher GHQ-28 scores for depressive symptoms in males compared to females. This was unexpected, considering that depression typically affects more females than males in the general population [76].

However, one possible explanation is that males may experience other problems, such as gender discrimination and role stereotyping [77]. Since discrimination, either direct or indirect, acts as a stressor, it can increase the risk of undesired mental health problems, such as psychological distress and depression [78].

Unfortunately, many studies exclusively use undergraduate students as a sample and usually compare them with the general population. Our sample comprised postgraduate students. However, more sex-related studies on postgraduate populations are needed for a safer interpretation of these results.

Mental health problems, such as depressive symptoms, anxiety symptoms, and suicidal ideation, are usually reported by people exposed to stressful life events [8, 9].

In a study conducted by Sokratous et al. [37] on Cypriot nursing undergraduate students, students who reported having experienced a significant number of stressful life events were more likely to experience depressive symptoms, similar to the findings reported by Reyes-Rodríguez et al. [79]. Moreover, the main causes of depressive symptoms have been proposed to be stressful life events, which are recognized risk factors for depression [12].

In the present study, approximately 57.8% of the participants reported vacations alone or with friends and beginning an undergraduate program at the university as the most stressful event. Cypriot postgraduate students mentioned minor financial problems, family get-togethers, vacations with parents, and minor legal violations as part of the top ten stressful life events, as well as a significant deterioration in the health of a close family friend. Moreover, most participants reported experiencing 3–6 stressful life events. Given that the existing literature supports the association between minor stressful life events and mental health distress, the above should be considered a substantial risk factor that increases an individual’s vulnerability to mental health problems [12, 72].

In a number of studies conducted among university students found a significant correlation between stressful life events in both personal and academic contexts, such as family separation, employment problems, educational satisfaction, issues with friends, sexual dysfunction, financial difficulties, and mental health [80, 81].

Moreover, in the study conducted by Sokratous et al. [37], a significant number of Cypriot students experienced minor life events, such as academic, social, and economical difficulties that raised their risk of developing depressive symptoms [37].

In the multiple linear regression analysis of the participants with the highest score on the LESS and those with the greatest number of stressful life events, males presented higher GHQ-28 scores than females.

This is in contrast to a study conducted by Sacco et al., who observed that stress from life events was more prevalent in females compared than in males [82].

Surprisingly, there were no statistically significant differences between the two groups in the relationship between mental health distress (GHQ-28 total score ≥ 24) and stressful life events, either by the total LESS score or the number of LESS events. A possible explanation for this finding is that several significant moderating factors have not been properly considered (e.g. inter-individual differences, especially psychological traits, as well as structural and social factors, such as generational and societal differences). Another possible explanation is that, as the literature suggests, the majority of people who undergo stressful situations do not experience long-lasting detrimental effects on their mental health, and some evidence suggests that low stress exposure may have protective effects [83].

Interestingly, individuals with a modest number of stressful life experiences who participated in various studies showed more beneficial effects (higher tolerances for pain and cardiovascular reactivity to laboratory stressors and less functional impairment) than individuals who did not report significant stressful experiences [84].

Finally, our study adds more evidence to the existing literature, providing new data on the association between stressful life events and mental distress clinically symptoms among a special population group, like the post graduate students. In the present study we measured the overall stressful experience in terms of 36 life events during the previous 12 months, rather than individual events. We showed that there is an association between clinically significant mental health symptoms and the number of reported events, and in fact, that the association persisted when the severity of these events was included in the measure. In addition, the present study supported further evidence regarding the validity of the GHQ-28 and LESS scales.

## Conclusion

There is an alarming prevalence of mental health distress among Cypriot university post graduate nursing students. Additionally, the number and the severity of stressful life events were related to the presence of mental distress clinical symptoms. There are important implications deriving from the findings of the present study in terms of identifying the most vulnerable students who are in need for psychological empowerment. Most importantly, in view of the relatively high mental distress symptoms among Cypriots post graduate university nursing students, there is a wider need to educate this population how to cope with stressors and mental distress symptoms, in order to achieve not only a better quality of life, but an elevated level of performance at individual and institutional level.

Postgraduate students in nursing and midwifery work in stressful circumstances; as a result, they must maintain good mental health to be able to conduct effective and safe clinical practice and provide high-quality nursing care. It has been proven that nurses who engage in postgraduate studies are more likely to have improved critical thinking and decision-making abilities, thus making such nurses a valuable frontline and basic labour force of the medical industry [85].

This study’s findings indicate that postgraduate students are susceptible to life burdens. Therefore, effective measures to prevent high levels of mental health problems from developing during their course of study must be ascertained, even at the postgraduate level.

The final recommendation is that nurse educators and nursing education

leaders communicate the insights collected from this study and provide the appropriate resources male students enrolled in nursing programs need. Educators and.

university counsellors should provide additional assistance to male students.

Students should be reminded of the range of student support services available by institutional counseling services should they experience poor mental health. This is an important objective; by doing so, the range of damaging mental and physical consequences to postgraduate students’ health can be minimised.

### Limitations

Our study has certain limitations. First, as our study was cross-sectional, information on stressful life events was gathered retroactively; thus, there may be inaccuracies or bias. Second, we examined only the life events included in the LESS and did not consider other severe stressors.

The data collection took place in university theatres; therefore, students who were absent on the day of data collection were excluded. Subsequently, it is possible that the observed prevalence of mental health the correlation between mental health symptoms and stressful life events may have been underestimated, as those who suffer from mental problems or psychological distress are less likely to attend classes regularly. Most importantly, the cross-sectional design of the study did not allow for any inference regarding the direction of the association between mental health symptoms and the frequency and intensity of stressful life events. For example, failing a course may be both the cause and the outcome of mental health problems. Similarly, cross-national comparisons are difficult because there is a need for multicentre international studies to explore the prevalence of mental health problems in student populations and in different cultures and settings using the same instruments and a standardised methodology.

Another limitation of the present study is its small sample size. The small sample size at the international level may limit the generalization of the results. At the national level, the sample was sufficient and it can attend representative; this is due to the fact that, at the time of data collection in our study, only one university (Cyprus University of Technology) offered a postgraduate degree in nursing. The results are worthable and remarkable for our institution and our counselling services by providing specific information concerning the profile of mental health status of our post graduate nursing students. However, the use of robust and appropriate tools (i.e. the GHQ-28 and student-specific LESS scale) to measure students’ mental health symptoms and stressful life events allows for a more accurate estimation of mental health problems and their correlation with stressful life events in the present population. Most importantly, in contrast to previous studies, the current study did not focus on particular events but assessed the extent to which the reported number of stressful life events and their severity were linked to mental health problems.

## Data Availability

The datasets generated and/or analyzed during the present study are not publicly available because the authors are currently working on them in order to prepare the final version of this manuscript. However, they are available from the corresponding author upon reasonable request.

## Abbreviations

GHQ: General Health Questionnaire
LESS: Life Events Scale for Students
CUT: Cyprus University of Technology
