# Correction to: Mental health status and stressful life events among postgraduate nursing students in Cyprus: a cross-sectional descriptive correlational study

**DOI:** 10.1186/s12912-023-01487-3

**Published:** 2023-09-19

**Authors:** Sokratis Sokratous, Giorgos Alexandrou, Rafailia Zavrou, Maria Karanikola

**Affiliations:** 1 Department of Nursing, Faculty of Health Sciences, Cyprus University of Technology, Limassol, Cyprus; 2 Mental health Services, Nicosia, Cyprus; 3 Mental health Services, Paphos, Cyprus

**Correction to: BMC Nursing (2023) 22:294**

10.1186/s12912-023-01463-x

Following publication of the original article [1], the authors reported that their family name and given name needed to be interchanged.

The correct author names have been updated in this Correction.

The original article [1] has been corrected.
